# Empowering medical students: bridging gaps with high-fidelity simulations; a mixed-methods study on self-efficacy

**DOI:** 10.1186/s12909-024-05996-w

**Published:** 2024-09-19

**Authors:** Pınar Daylan Koçkaya, Tuncay Müge Alvur, Orhan Odabaşı

**Affiliations:** 1 Division of Basic Medical Sciences, Department of Medical Education, Kocaeli University Faculty of Medicine, Kocaeli, Turkey; 2 Division of Internal Medical Sciences, Department of Family Medicine, Kocaeli University Faculty of Medicine, Kocaeli, Turkey; 3 Basic Sciences Division, Department of Medical Education and Informatics, Hacettepe University Faculty of Medicine, Ankara, Turkey

**Keywords:** Advanced Life Support, Cardiopulmonary resuscitation, Medical education, Simulation training, Self efficacy

## Abstract

**Background:**

High-fidelity simulations play a crucial role in preparing for high-mortality events like cardiopulmonary arrest, emphasizing the need for rapid and accurate intervention. Proficiency in cardiopulmonary resuscitation(CPR) requires a strong self-efficacy(SE); training for both is crucial. This study assesses the impact of Advanced Life Support(ALS) simulation on SE changes in final-year medical students.

**Methods:**

This mixed-methods prospective simulation study involved medical students in emergency medicine internships, examining self-efficacy perceptions regarding ALS technical skills(ALS-SEP). A comparison was made between students who underwent scenario-based ALS simulation training and those who did not. Competencies in chest compression skills were assessed, and the concordance between ALS-SEP scores and observed CPR performances were evaluated. Focus group interviews were conducted and analyzed using content analysis techniques.

**Results:**

The study involved 80 students, with 53 in the experimental group(EG) and 27 in the control group(CG). The EG, underwent simulation training, showed a significantly higher ALS-SEP change than the CG(*p* < 0.05). However, there was low concordance between pre-simulation SEP and actual performance. Compression skills success rates were inadequate. Qualitative analysis revealed main themes as"learning“(32.6%), “self-efficacy“(29%), “simulation method“(21.3%), and “development“(16.5%).

**Discussion:**

Post-simulation, students reported improved SEP and increased readiness for future interventions. The findings and qualitative statements support the effectiveness of simulation practices in bridging the gap between SEP and performance. Utilizing simulation-based ALS training enhances learners’ belief in their capabilities, raises awareness of their competencies, and encourages reflective thinking. Given the importance of high SEP for ALS, simulation trainings correlating self-efficacy perception and performance may significantly reduce potential medical errors stemming from a disparity between perceived capability and actual performance.

## Introduction

Sudden cardiac arrest (SCA) remains a significant factor in global mortality rates and poses a considerable public health problem, even with many advancements and ongoing efforts directed toward the prevention and treatment of SCA. SCA is the third most common cause of death in Europe [1].

Simulation training plays a significant role in cardiopulmonary resuscitation (CPR) training. Both high- fidelity and low-fidelity simulation modalities facilitate contextual learning for students with different learning levels and methods. They provide an opportunity to integrate both technical and non-technical skills. Simulation enables learners to acquire the necessary competencies to effectively manage human factors, particularly in CPR scenarios. Profound learning occurs during the reflective phase of debriefing [2]. Simulation-based training for resuscitation is reported to be highly effective. Evidence indicates that high- fidelity simulations enhance learning when applied under appropriate conditions [3]. Simulation-based CPR training outperforms training methods without simulation involvement, with topics such as teamwork included. Moreover, the effectiveness of simulation training increases with a design that incorporates structured feedback opportunities [4].

Self-efficacy (SE), which was first defined by Bandura [5], refers to an individual’s self-judgment of possessing the necessary characteristics to organize and execute actions required for successfully performing a specific duty or task. SE is also defined as a cognitive perception factor that is affected by individual behaviors. In the context of students, SE encompasses their assessment of proficiency in the training activities encountered throughout their educational journey, aligning with their expectations. Notably, SE has been identified as a crucial determinant for achieving success in psychomotor skills [6].

Studies examining the relationship between health simulation and self-efficacy consistently indicate that various simulation training programs enhance performance-related self-efficacy [7–13]. However, it is crucial to consider the alignment between clinicians’ perceived abilities or self-efficacy and their actual proficiency levels. Clinicians with high self-efficacy perception but low performance may have a detrimental impact on patient outcomes, particularly in critical conditions such as resuscitation [14]. The debriefing step, which allows for the evaluation of individual performance during the scenario using simulation, can increase self- efficacy perceptions related to performance, specifically through reflection. This process can effectively eliminate conditions that pose a risk to the patient. Self-efficacy perception has a significant impact on the cognitive, emotional, psychomotor, and social aspects of resuscitation capability. Even clinicians who possess knowledge and proficiency in their techniques cannot successfully apply these skills without a strong belief in their self-efficacy regarding the practice itself. The importance of applications aimed at increasing self-efficacy perception in CPR training cannot be overstated [15].

Enhancing students’ self-efficacy perception is of significance because it contributes to academic achievement, training interest, and the intended outcomes of the training. To promote an increase in students’ self-efficacy perception, trainers are advised to establish clear, specific, and attainable learning objectives, along with challenging learning objectives that require effort. Additionally, providing accurate and equitable feedback, fostering the development of an accurate self-efficacy perception, and incorporating peer experiences into the training are recommended strategies [16].

This study aimed to examine whether scenario-based advanced life support simulation training affects the self-efficacy perception of intern students in their final year of medical school. This study assessed the relationship between students’ technical CPR abilities, self-efficacy perception, and performance, as well as the impact of gender on these abilities and perceptions. Additionally, the study sought to explore the students’ experiences and opinions regarding the simulation training in which they participated.

This study was previously presented at the National Medical Education Congress (UTEK22) 12th Annual Scientific Assembly on May 20, 2022.

## Methods

### Study design and sample size

This prospective study was conducted at Kocaeli University, School of Medicine, between February and October 2020. This study aimed to evaluate the effect of scenario-based simulation on the self-efficacy in relation to their technical skills in ALS. The study followed a mixed research design, incorporating both quantitative and qualitative elements. The quantitative phase involved a semi- experimental approach with both experimental and control groups. The qualitative phase involved content analysis of the focus group interviews. All senior medical students were eligible to participate in this study. Only students who had completed the face-to-face Anesthesiology and Reanimation internship, the ALS course, and had attended ALS with real patients, but had not previously participated in the simulation, were included.

Based on the power analysis conducted using the G*Power program, it was determined that a minimum of 26 individuals per group (experimental and control) would be required, assuming an effect size of 0.80, α = 0.05, and Power (1-β) = 0.80. For this study, 80 students voluntarily participated, with 53 assigned to the experimental group and 27 to the control group, which are higher than the calculated minimum number of each group depending on power analysis. Furthermore, focus group interviews were conducted with 11 students. The control group exclusively comprised students who proceeded with their regular emergency medicine internship, whereas the experimental group students actively engaged in scenario-based simulation intervention as part of their internship experience.

The research was conducted in three stages: preparation, pilot testing, and simulation. During the preparation stage, data collection tools for the research were developed. The ALS scenario was developed based on the expertise of a specialist group comprising emergency medicine specialists, national ALS instructors, and paramedics from national emergency health services, 112. To ensure usability and evaluate the technical aspects of the simulation training, two pilot tests were conducted to assess the forms and technical features.

### Simulation

The high-fidelity simulation stage was conducted at the Kocaeli University Simulation Center (KOUSIM), where video recordings were obtained. The simulation involved teams of three students, each equipped with CPR feedback wristbands. In the scenario outlined in the study, the students were assigned the task of providing intervention for a 55-year-old male patient who suffered cardiac arrest and was diagnosed with chronic obstructive pulmonary disease. The patient presented to the hospital emergency department with respiratory distress. A panicked family member provided the students with information about the patient’s deteriorating condition. Upon initial contact with the students, the patient was unresponsive. During the events, the observed arrest rhythms initially showed asystole, followed by ventricular fibrillation. Before the simulation, the students assessed their self-efficacy perceptions in various areas of ALS technical skills, such as compression quality, airway management, medication administration, and defibrillation skills. They were instructed to perform continuous chest compression for a duration of 2 min and to execute all the necessary skills at least once. The scenario was designed to end with asystole unless a shockable rhythm was identified.

The researchers assessed the technical skills of the students using a mid-fidelity ALS simulator, and then reviewed video recordings during the debriefing session.

### Data collection

In the research, various forms were utilized, namely the Introductory Information Form, ALS Technical Skills Self-Efficacy Perception (ALS-SEP) Form (Table 1), ALS Technical Skills Assessment Tool (Table 2), Evaluation of Simulation Feedback Form (Table 3), and Focus Group Interview Form.

**Table 1:** Advanced Life Support Technical skills Self-Efficacy Perception Form

Please assess your self-efficacy perception for each step of the cardiopulmonary resuscitation (CPR) technical skills outlined in the following articles. Use a scale from 1 to 10 to indicate your level of agreement with each proposition. Self-efficacy refers to an individual’s belief in their capabilities to perform a given task. Score 10 if you strongly agree with the proposition as “I certainly do,” and 1 if you strongly disagree with the proposition as “I certainly cannot do.” Thank you for your attendance and support.											
	For the cardiopulmonary resuscitation										
No	Proposition	10	9	8	7	6	5	4	3	2	1
1	I can perform effective cardiac compression (5–6 cm depth, 100–120 compression rate/min).										
2	I can maintain an open-air passage										
2a	I can respirate the patient with a bag valve mask-ambu.										
2b	I can perform endotracheal intubation.										
3	I can establish vascular access.										
4	I can defibrillate the patient.										
4a	I can recognize the rhythm on the monitor.										
4b	I can set up the required Joule.										
5	I can administer required medications.										

**Table 2:** Advanced Life Support Technical Skills Assessment Tool

	ADVANCED LIFE SUPPORT TECHNICAL SKILLS ASSESSMENT TOOL Date:				S1	S2	S3
	Criteria	0-Insufficient/ not observed No administration, wrong administration of the step, or inability to administer it for the required time	1-Needs to be developed Partly correct administration of the step or administering it for the required time but the presence of insufficiencies and/or need for the trainer’s help or reminder	2-Sufficient/ observed: The correct administration of the step in the correct order and without hesitation and need of help			
1	Can perform effective cardiac compression. (5- 6 cm depth, 100- 120 compression rate/min).	The compression rate is incorrect, and the compression depth is incorrect.	(a) The compression rate is accurate, but the depth of compression is incorrect. (b) The compression rate is incorrect, but the depth of compression is accurate.	Both compression depth and rate were applied accurately.			
2	Able to maintain airway opening	Did not check the airway opening. Did not apply airway	(a) Applied airway opening maneuver after checking the airway opening. Did not correctly apply airway - never applied airway (b) Did not check airway opening, did not apply airway opening maneuver Correctly applied airway.	Checked airway opening and applied airway opening maneuver. Correctly applied airway			
2a	Able to respirate the patient with a bag valve mask (BVM)	Incorrectly held the BVM Could not ventilate the patient	Held the BVM with the correct maneuver (C-E) Could not provide enough ventilation	Held the BVM with the correct maneuver and provided enough ventilation without air leakage			
2b	Able to perform endotracheal intubation.	Could not perform endotracheal intubation	Could not serially perform intubation, the sound of tooth trauma is heard from the simulator.	Intubated in a serial manner.			
3	Able to establish vascular access	Could not establish vascular access	Could establish vascular access after repetitive trials.	Could quickly establish vascular access			
4	Able to defibrillate	Could not correctly place the defibrillator paddles	Was able to accurately position the defibrillator paddles and did not adhere to the safety defibrillation protocols.	Was able to accurately position the defibrillator paddles and followed the safety protocols for defibrillation.			
4a	Able to recognize the rhythm on the monitor	Did not recognize the rhythm	Understood that it is a shockable rhythm Could not diagnose VF	Understood that it is a shockable rhythm and diagnosed VF			
4b	Able to set up the required Joule	Configured an incorrect joule energy setting for both defibrillators without discerning the distinction between monophasic and biphasic defibrillator requirements.	Configured the energy dose for the biphasic defibrillator.	Accurately configured the necessary energy dose for the monophasic defibrillator.			
5	Able to administer medications	Administered drugs in an incorrect order and with inaccurate dosages.	a. Administered the drug doses inaccurately, but in the correct sequence. b. Administered the accurate drug dosage, but not in the correct order.	Administered the medications accurately, adhering to the correct timing and frequency.			

**Table 3:** Simulation Feedback Form

Proposition	Totally Agree	Agree	I can’t decide	I don’t agree	I never agree
1. Sufficient information was made by the simulation practice.					
2. The simulation scenario was designed to be realistic.					
3. I felt comfortable during the simulation practice.					
4. We were able to conduct an effective teamwork during the simulation application.					
5. The discussion session at the end of the simulation was beneficial.					
6. I gained an awareness of my resuscitation skills level after the simulation practice.					
7. I realized the importance of teamwork.					
8. After the simulation, I became aware of my leadership skills in the resuscitation					
9. I would like to participate in an advanced life support application organized with another scenario.					
10. The simulation can enhance my medical skills.					
11. The simulation training should play a part in the practices of medical education which especially involve teamwork and at which leadership characteristics are demonstrated.					
12. After this simulation, I can provide advanced life support more confidently in my future professional career.					
13. I am satisfied with participating in the advanced life support simulation					
14. Please evaluate this simulation on a scale of 1 (Very Poor) to 5 (Excellent)……………….					

The researchers developed the ALS-SEP form through consultation with physicians, paramedics, and national ALS trainers. They also considered the technical skills required during ALS according to ERC and AHA guidelines [17–18]. These skills encompass chest compression rate and depth, airway and ventilation management, establishing vascular access, defibrillation, and drug administration. The form consists of nine criteria To assess these items, a 100-mm Visual Analog Scale (VAS) was used, which has been validated for evaluating self-efficacy in resuscitation skills [19]. The Visual Analog Scale (VAS) consists of a fixed line measuring 100 mm between two opposing adjectives (e.g., very low - very high). Participants mark a point on the line representing their perceived condition. The distance between this mark and the starting point was measured to assess the participant’s self-efficacy level. In the VAS scoring of the ALS-SEP Form, a score of 10 indicates “I certainly can do it,” while a score of 1 signifies “I certainly cannot do it.” The ALS-SEP form was administered as a pre- test (PET) and post-test (POT) to assess the participants’ initial conditions in both the experimental and control groups and their conditions at the end of the internship. PET was implemented during the first quarter of the emergency medicine internship, while PEA was implemented at the end of the internship.

Compression skills were evaluated using a CPR feedback wristband that measures compression depth and rate. The researchers observed and evaluated the students’ performance using the ALS Technical Skills Assessment Tool. The correlation between the ALS-SEP scores and observed performances was evaluated. Feedback was collected after the simulation using a 5-point Likert scale, which included 14 statements and 1 open-ended question. Additionally, focus group interviews were conducted online following the internship.

### Data analysis

Statistical analysis of quantitative data was conducted using IBM SPSS 20.0 (IBM Corp., Armonk, NY, USA) software package. The normal distribution assumption was assessed using the Kolmogorov- Smirnov and Shapiro- Wilk tests. Numerical variables were presented as either mean ± standard deviation or median (25th-75th percentile), whereas categorical variables were presented as frequency (percentage). Due to the inability to assume a normal distribution, non-parametric methods were used for statistical analysis. The Mann- Whitney U test was used to assess differences between groups, and the Wilcoxon signed-rank test was used to examine differences between paired samples. Kappa statistics were used for compatibility analysis. A significance level of *p* < 0.05 was considered statistically significant for two-tailed hypothesis tests.

The ALS-SEP was evaluated for each skill individually, analyzing the change in self-efficacy perception. The average score of all self-efficacy ratings was calculated as the “combined self-efficacy score (CSES)” for the ALS skill, which was defined as a set of sequential procedures within the chain of survival framework [17–18].

The CPR feedback wristband collected data on achieving a compression depth of 5–6 cm (± 2 mm) and a compression rate of 100–120/min. Additionally, the device provided success percentage ratios for both depth and rate parameters during compression.

The researchers observed and assessed the students’ performance, assigning scores of 0 points for inadequate or unobserved performance, 1 point for performance needing improvement, and 2 points for adequate performance. The concordance between the ALS-SEP scores and observed performances was evaluated. To facilitate appropriate analysis between the observer’s 3-point scoring and the students’ 10- point VAS scoring, the VAS scoring was categorized based on the expert opinion of ALS instructors. In this categorization, the 1-2-3-4 lines on the VAS scale corresponded to 0 points, the 5-6-7 lines corresponded to 1 point, and the 8-9-10 lines corresponded to 2 points. Kappa concordance statistics were used to assess the agreement between the two scoring methods.

The content analysis method was used to evaluate the focus group interviews using recorded interview data. The codes were derived from the interview data and organized to identify themes, as well as to explore concepts and relationships. Categorical data is presented in terms of frequency and percentage.

### Limitations

This study had some limitations. First, the randomization process for the volunteers at the beginning of the study had to be altered because of the mandatory interruption in the training schedule caused by the COVID-19 pandemic. As a result, the first volunteer group was designated as the control group, whereas the experimental group comprised students who were able to start face-to-face training. The pandemic has had some impact on the conduct and logistics of this study. The initial randomization process was disrupted due to mandatory interruptions in the training schedule, resulting in a smaller control group and a larger experimental group. The power analysis verified that the calculated sample sizes were adequate to identify significant effects, even considering the challenges encountered. Despite the smaller control group, the experimental group size was larger than the calculated minimum, providing a stronger basis for statistical analysis and increasing the reliability of our findings. Following the face-to-face simulation training and the overwhelmingly positive feedback it received, the simulation became a mandatory component of the curriculum. This development led to the design and opening of a simulation center at our faculty, equipped with a greater number of new high-fidelity simulators, making it impossible to maintain a control group in subsequent iterations of the study. The larger size of the experimental group was facilitated by the logistics of implementing the new simulation-based training program, which allowed for a more comprehensive assessment of the simulation’s impact on self-efficacy and performance.

It’s important to note that the participants in the experimental group, who received the simulation training post-isolation, might have experienced heightened levels of self-efficacy. This increase in self-efficacy is primarily attributed to the intensive nature of the simulation training itself, which was designed to enhance ALS skills and self-efficacy. Additionally, the unique context of the pandemic may have influenced the students’ overall resilience and adaptability, potentially contributing to their readiness to engage with and benefit from the simulation training. However, the core enhancement in self-efficacy is directly linked to the structured, high-fidelity simulation program implemented. Additionally, this research was conducted in a single center.

## Results

In the control group, there were 12 women (44.4%) and 15 men (55.6%), while in the experimental group, there were 35 women (66%) and 18 men (33.9%). There was no statistically significant difference in gender distribution between the groups (*p* = 0.054, *p* > 0.05). The average age of participants in the control and experimental groups was 23.74 ± 0.44 and 23.58 ± 0.49 years, respectively. No significant difference was observed (*p* > 0.05).

None of the participants in either the control or experimental groups had previously received high-fidelity simulation training. An evaluation was conducted based on their participation in performing specific tasks during the ALS training. All participants had experience performing tasks such as “cardiac compression” and “ventilation of intubated patients using a bag-valve mask” at least once.

### Quantitative results

**Fig. 1:**
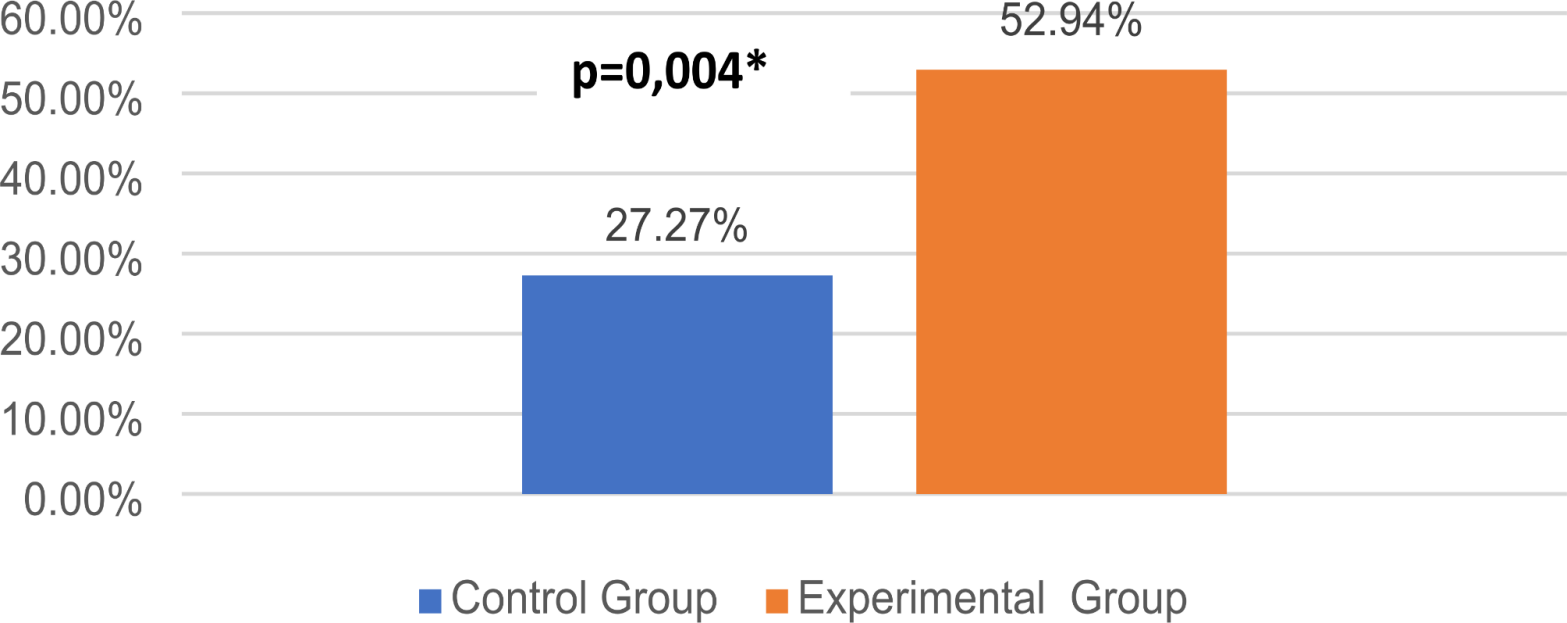
Comparison of the percentage change in the combined self-efficacy scores between the pre-test and post-test

When comparing the results of PET for ALS-SEP between the control and experimental groups, no statistically significant difference was found, except for the adjustment of the required energy dose for defibrillation (*p* > 0.05) (Table 4). The POT results regarding ALS-SEP were evaluated for both groups. Statistical significance was observed for several topics, including maintaining airway opening, bag valve mask usage, endotracheal intubation, and drug administration (*p* < 0.05) (Table 5). The experimental group showed a higher increase in the percentage of change in CSES (52.94%) than the control group (27.27%), and this increase was statistically significant (*p* < 0.05) (Fig. 1). The results of the increase in percentage in both groups were significantly different for specific questions related to skills (*p* < 0.05) (Table 6). The percentage of change in CSES was measured as 44.64 (28.86–78.57) and 44.82 (23.98–64.11) in women and men, respectively, with no statistically significant difference between genders (*p* > 0.05). According to CPR wristband results, the percentage of students reaching the expected compression depth was 17.24% 17.24 ± 23.18%, and their ability to compress at the expected rate interval was 29.90% 29.90 ± 37.36% (Table 7). The number of students who achieved at least 80% of the expected depth and rate was 2 (3.7%) and 13 (24.52%), respectively.

**Table 4:** Comparison of students’ pre-test self-efficacy perceptions of Advanced Life Support Technical skills

Question Number	Statements	Control (*n* = 27) Median (IQR)	Experimental (*n* = 53) Median (IQR)	*p*- value *
Question 1	I can perform effective cardiac compression (5–6 cm depth, 100–120 compression rate/min).median (Q1-Q3)	8,00 (6,00–9,00)	8,00 (6,00–8,00)	0,179
Question 2	I can maintain an open-air passage median (Q1-Q3)	7,00 (5,00–8,00)	6,00 (5,00–7,00)	0,164
Question 2a	I can respirate the patient with a bag valve mask median (Q1-Q3)	9,00 (8,00–10,00)	8,00 (7,00–9,00)	0,057
Question 2b	I can perform endotracheal intubation median (Q1-Q3)	5,00 (3,00–7,00)	4,00 (2,50 − 6,00)	0,332
Question 3	I can establish vascular access. median (Q1-Q3)	7,00 (5,00–8,00)	6,00 (4,00–8,00)	0,295
Question 4	I can defibrillate the patient median (Q1-Q3)	6,00 (2,00–7,00)	5,00 (2,00–6,50)	0,442
Question 4a	I can recognize the rhythm on the monitor. median (Q1-Q3)	7,00 (4,00–8,00)	6,00 (4,00–7,00)	0,244
Question 4b	I can set up the required Joule. median (Q1-Q3)	6,00 (3,00–8,00)	4,00 (2,00–6,00)	*0,022
Question 5	I can administer the necessary medications. median (Q1-Q3)	5,00 (3,00–7,00)	4,00 (3,00–7,00)	0,611

*Mann-Whitney U, Q1:25th. percentile Q3: 75th percentile, ** *p*-value < 0,05 is statistically significant

**Table 5:** Comparison of students’ post-test self-efficacy perceptions of Advanced Life Support Technical skills

Question Number	Statements	Control (*n* = 27) Median (IQR)	Experimental (*n* = 53) Median (IQR)	*p*- value *
Question 1	I can perform effective cardiac compression (5–6 cm depth, 100–120 compression rate/min).median (Q1-Q3)	9,00 (8,00–10,00)	9,00 (8,00–10,00)	0,374
Question 2	I can maintain an open-air passage median (Q1-Q3)	8,00 (7,00–9,00)	9,00 (8,00–10,00)	**0,014
Question 2a	I can respirate the patient with a bag valve mask median (Q1-Q3)	10,00 (9,00–10,00)	9,00 (8,50 − 10,00)	**0,028
Question 2b	I can perform endotracheal intubation median (Q1-Q3)	7,00 (5,00–8,00)	8,00 (7,00–9,00)	**0,004
Question 3	I can establish vascular access. median (Q1-Q3)	9,00 (7,00–10,00)	8,00 (7,00–9,00)	0,195
Question 4	I can defibrillate the patient median (Q1-Q3)	9,00 (6,00–10,00)	9,00 (8,00–10,00)	0,259
Question 4a	I can recognize the rhythm on the monitor. median (Q1-Q3)	8,00 (7,00–10,00)	8,00 (7,00–9,00)	0,963
Question 4b	I can set up the required Joule. median (Q1-Q3)	9,00 (8,00–10,00)	9,00 (8,00–10,00)	0,588
Question 5	I can administer the necessary medications. median (Q1-Q3)	8,00 (7,00–9,00)	9,00 (8,00–10,00)	**0,005

*Mann-Whitney U, Q1:25th. percentile Q3: 75th percentile, ** *p*-value < 0,05 is statistically significant

**Table 6:** Comparison of the percentage change in students’ self-efficacy perceptions of Advanced Life Support Technical skills

Variables	Control (*n* = 27) Median (IQR) %	Experimental (*n* = 53) Median (IQR) %	*p*- value*
Change Percentage of Combined self-efficacy score between pre-test and post-test, median (Q1-Q3)	27,27 (9,58 − 54,54)	52,94 (29,78–78,17)	**0,004
Change Percentage of Question 1 between pre-test and post-test, median (Q1-Q3)	12,50 (0,00–33,33)	14,28 (11,11–41,42)	0,348
Change Percentage of Question 2 between pre-test and post-test, median (Q1-Q3)	11,11 (0,00–50,00)	50,00 (25,00-100,00)	**0,000
Change Percentage of Question 2a between pre-test and post-test, median (Q1-Q3)	0,00 (0,00–12,50)	12,50 (0,00–25,00)	0,386
Change Percentage of Question 2b between pre-test and post-test, median (Q1-Q3)	16,66 (0,00-100,00)	100,00 (28,57–200,00)	**0,001
Change Percentage of Question 3 between pre-test and post-test, median (Q1-Q3)	12,50 (0,00–33,33)	28,57 (5,55–55,00)	0,271
Change Percentage of Question 4 between pre-test and post-test, median (Q1-Q3)	28,57 (12,50–125,00)	66,66 (30,95–241,66)	**0,018
Change Percentage of Question 4a between pre-test and post-test, median (Q1-Q3)	14,28 (0,00–80,00)	40,00 (12,50–112,50)	0,069
Change Percentage of Question 4b between pre-test and post-test, median (Q1-Q3)	33,33 (0,00-150,00)	133,33 (42,85–375,00)	**0,003
Change Percentage of Question 5 between pre-test and post-test, median (Q1-Q3)	60,00 (12,50–150,00)	100,00 (41,42–233,33)	0,116

*Mann-Whitney U, Q1:25th. percentile Q3: 75th percentile, ** *p*-value < 0,05 is statistically significant

*Mann-Whitney U *p-value < 0,05 is statistically significant

**Table 7:** Success percentages of CPR Compression rate and depth

	Mean ± Standard Deviation
Percentage of success for depth (%) (n:53)	17,24 ± 23,18
Percentage of success for compression rate (%) (n:53)	29,90 ± 37,36

The agreement between the performance scores assigned by the observers and the self-efficacy perceptions reported by the students was evaluated using Kappa concordance statistics, indicating a low level of agreement (Kappa < 0.2). No significant correlation was observed between the students’ performance and their self-efficacy perceptions (Table 8).

**Table 8:** Results of Kappa Concordance statistics between self-efficacy perceptions and performances of the Experimental Group

Question number	Kappa value*	Standard error	%99 Confidence interval	
			lower limit	upper limit
Question 1	,001	,056	1,000	1,000
Question 2	-,009	,089	1,000	1,000
Question 2a	,023	,041	,000	,000
Question 2b	,151	,103	,105	,121
Question 3	-,004	,109	1,000	1,000
Question 4	,189	,083	,052	,064
Question 4a	,068	,042	,209	,231
Question 4b	-,035	,035	1,000	1,000
Question 5	-,024	,083	,859	,877

*Kappa value < 0.2: No agreement

### Qualitative results

The main topics of discussion among the students revolved around their perceptions of simulation, their experiences with simulation training, the benefits they gained from simulation, and their recommendations. After analyzing the data, four main themes emerged, namely “learning,” “self-efficacy,” “simulation method,” and “development.” The analysis revealed that the learning theme represented 32.6% of the opinions, followed by the “self-efficacy” theme at 29%, the “simulation method” theme at 21.3%, and the development theme at 16.5% (Table 9).

**Table 9:** Focus Group Codes and themes

Themes	Unit	% (*n*:309)	Codes
Learning	101	32.6	Learning the correct intervention at CPR
			The idea that the learned skills will be permanent
			Learning by experiencing and practically
			Opportunity to make a self-evaluation
			Its teaching nature
Self-efficacy perception	91	29	The perception that he or she was more sufficient than he or she was before the simulation
			The idea that he or she can intervene more accurately in the future
			Recognition of individual insufficiencies and inexperience
			Managing CPR alone
Simulation method	66	21.3	Obtaining a video recording and watching it
			Perception of fidelity
			Effect of the debriefing session
			Feelings
Development	51	16.5	Need of simulation training in the previous periods
			Need of more scenarios
			Need to develop the technological background
Total	309	100	

The content analysis method was used to evaluate the focus group interviews. Categorical data is presented in terms of frequency and percentage. n: 11 students

Regarding the “learning” theme, the students expressed that the knowledge and skills they gained through simulation training were long-lasting. They emphasized that the training was instructive and informative, especially in relation to CPR, and that they learned effectively by actively participating in simulation scenarios. They also mentioned that self-evaluation helped them recognize their efficiency and learn about appropriate interventions (Table 10).

**Table 10:** Distribution of the codes in the learning theme

Theme		Codes	Unit	% (*n*: 101)
Learning		Correct intervention recognition and learning in CPR	40	39.60
		Permanence of learned skills	17	16.83
		Experiential and practical learning	14	13.86
		Opportunity for self-evaluation	14	13.86
		Instructive nature	16	15.84
		Total	101	100
Selected Quotes for Learning Theme	Student 1	“The interweaving of theory and practice in this exercise brought about a more sophisticated and highly informative experience for me. In fact, it proved to be an exceptionally educational endeavor.”		
	Student 6	“Teacher, I believe that I learn through hands-on experience even if it doesn’t match one-to-one. What I mean is, there are differences between a real human and a physical model, of course. But I’ve learned how the process works, how to manage stress, at least by following the procedure’s sequence, by actually experiencing and performing it to understand how I should apply it.”		
	Student 7	““Regarding reviewing the recording, one might believe they are performing chest compressions very rapidly, for instance. However, upon watching the video, we came to realize how comparatively slower our actual pace was. This revelation proved to be impactful.”		
	Student 10	““In my opinion, people learn more as they experience; this practice taught us through experiences. While performing CPR, I feel more competent, especially through the bracelet measurements, I understood it better.”		
	Student 11	““What we knew and didn’t know became evident. It wasn’t like an exam. We truly saw where we stood. I could analyze my own actions, for example, both our individual performance and the team dynamics were visible, and we understood the importance of teamwork. I found it highly valuable, and I learned a lot.”		

In relation to the theme of “self-efficacy perception”, the students compared their competence before and after the simulation training. A significant portion of the quotes were reported as “The perception of being more competent compared to before the simulation” (31.87%). They believed that the experience improved their ability to accurately intervene in future situations and increased their confidence in managing ALS. They recognized their personal limitations and lack of expertise, which contributed to the growth of their self-efficacy (Table 11).

**Table 11:** Distribution of the codes in the theme of self-efficacy perception

Theme		Codes	Unit	% (*n*:91)
Self-efficacy perception		The perception of being more competent compared to before the simulation	29	31.87%
		The belief in the ability to intervene more accurately in the future	22	24.18%
		Recognition of individual deficiencies and inexperience	26	28.57%
		Ability to manage CPR alone	14	15.38%
		Total	91	100
Selected Quotes for Learning Theme	Student 1	“Now I do things in some way with more confidence in myself, and I can say yes, these are correct. I can believe in the things I do and say I can stand behind them now.”		
	Student 3	“For the first time, ALS management was entirely left to us, and we saw the benefits of this. There was a chance to see our mistakes. It’s great, of course, to be more competent.		
	Student 5	“I received CPR training during my anesthesia internship, and I had learned it quite well back then. But I don’t remember it being etched in my mind like this. That’s why it was truly enjoyable and highly instructive for me, as I mentioned before. You know, I’ve begun to feel adequately equipped, like a physician who has learned something useful and can contribute to something valuable. Thank you.”		
	Student 8	“So, in this simulation, we not only witness our own development but also feel more self-assured. Because when I try things on a model and then apply them on a patient, I become more confident. My self-confidence also grows. However, when I do it on an actual patient, I feel a bit less confident and inexperienced. That’s why such a practice is absolutely necessary. I believe it’s very beneficial for us.”		
	Student 9	“While doing it, we also realized that we had a lot of shortcomings; we were kind of just looking at each other in the group. We saw that there were quite a few deficiencies; it was good to see them. Now, I think we can act more competently.”		
	Student 10	“The gain from this training for me is the confidence that I can do it on my own. Instead of wondering if I would do it or not, now I instantly recall what I need to do. My self-confidence has increased, and I’m less anxious when encountering a patient. All of these have become lasting changes in me.”		
	Student 11	“I believe that my performance will be better after this practice because we hadn’t had the chance to experience it alone before. So, I definitely think I’ll intervene better now. Also, I saw my mistakes and believe I won’t repeat them. I gained more confidence in myself when it comes to actually doing it. I have a clear sense of ‘I can do it.‘”		
	Student 11	“I performed the CPR as aware of many things. In the emergency room, things happen quickly; it’s crowded, and we just assist. Here, being on our own, I understood better. I believe I would perform more confidently even on my own.”		

The students primarily focused on the perception of fidelity within the theme of “simulation.” They shared their views on the significance of reviewing recordings during debriefing sessions and gaining the skills and knowledge they lacked (Table 12). Furthermore, they highlighted the importance of simulation training in earlier medical education semesters and expressed a wish for a wider range of scenarios in the future (Table 13).

**Table 12:** Distribution of the codes in the theme of Simulation Method

Theme		Codes	Unit	% (*n*:66)
Simulation method		Obtaining a video recording and watching it	13	19.70
		Perception of fidelity	22	33.33
		Feelings	20	30.30
		Effect of the debriefing session	11	16.67
		Total	66	100
Selected Quotes for Simulation Method Theme	Student 2	“First of all, the simulation was truly realistic. The role of the patient’s relative was very helpful for me, to get into the mode there. It really was one of the most beneficial practices in these six years of education.”		
	Student 3	“Seeing the recording was very different. I didn’t believe it even when the instructors told me. I used to think that I was doing enough. I used to say that back then, but when I watched it on the camera, I understood what I couldn’t do. It was even more impactful than the instructors for me.”		
	Student 7	“The presence of the patient’s relative inside increased the realism, and your not intervening as much as possible made us feel like we were there on our own. It was very realistic.”		
	Student 10	“It was very close to reality, and we were making decisions on our own. In the emergency room, there are always residents. Here, we were alone, just like we will be when we graduate and work on our own.”		

**Table 13:** Distribution of the codes in the theme of development

Theme		Codes	Unit	% (*n*:51)
Development		Previous periods’ need for simulation training	18	35.29
		Need for more scenarios	18	35.29
		Need for technological infrastructure development	15	29.41
		Total	51	100
Selected Quotes for Development Theme	Student 1	“It could have been more technological. Of course, when technology and resources allow, it can be further developed.”		
	Student 6	“I certainly think that this training should be provided every year regularly starting from semester one.”		

In response to the question presented in Table 3, “After this simulation, I can provide advanced life support more confidently in my future professional career,” 98.11% of the students expressed agreement. Additionally, all the students conveyed satisfaction with their involvement in the ALS simulation, rating the training an average of 4.88 out of 5 points.

## Discussion

Scenario-based high-fidelity simulation training offers valuable opportunities for individual, team, and interdisciplinary training. It serves as a platform for evaluating decision-making processes and fostering continuous improvement. In addition, it supports learning by providing reflective and analytical steps that facilitate individual development [20]. Interactive training methods such as simulation training, which include accurate feedback and correction of errors, can improve the relationship between self-efficacy and competence. Performance is influenced by a complex interplay of preparedness, self-efficacy, and acquired knowledge and skills. The students’ perception of self-efficacy, which can be developed through reflection during simulation training, could significantly influence their professional performance and motivation to enhance it. According to Bandura’s theory [21], students with high self-efficacy tend to achieve more successful, effective, and efficient outcomes than those with low self-efficacy. Students with high self- efficacy demonstrate increased effort and motivation when faced with challenges. Moreover, the level of self- efficacy can influence task selection, motivation, and willingness to perform, as students with low self- efficacy may be more prone to avoid or withdraw from tasks due to fear [22].

Various methods, such as low or high fidelity models, virtual and web-based applications, or scenario-based simulation, can be used to teach CPR skills. Regardless of the chosen method, it is essential to have effective feedback mechanisms that provide accurate assessments of student performance [2].

The perception of high self-efficacy in performing CPR is closely linked to the delivery of high-quality CPR [23]. Therefore, ALS training that focuses on improving both CPR skills and self-efficacy can be highly beneficial. Recently, there has been an increasing preference for training that focuses on fundamental skills required for performing high-quality CPR, as well as scenario-based simulation training that closely replicates real-life situations. Evidence supporting the use of simulation methods in enhancing self-efficacy for various skills exists, including CPR. A systematic review has demonstrated that simulations with high fidelity, conducted under accurate conditions, facilitate the learning of CPR skills [3].

Another systematic review and meta-analysis have demonstrated that simulation-based CPR training, which includes teamwork and structured feedback, is superior to training without simulation in terms of knowledge and skill acquisition [4]. Moreover, high-fidelity simulation training is considered to be more effective than low-fidelity CPR model training for teaching high-quality CPR to medical students [24]. There is increasing evidence suggesting that students educated with simulation-based CPR curricula exhibit better proficiency in learning ALS compared with those following the traditional curriculum [25]. In addition, simulation has been found to significantly enhance the quality of care provided by assistants during real ALS cases, suggesting that it can complement traditional procedural training methods [26].

Although clinicians possess knowledge and expertise in their techniques, their ability to effectively apply these skills is associated with a strong self-efficacy belief in their abilities. Healthcare professionals with a high perception of self-efficacy in CPR are expected to demonstrate successful CPR performance using their acquired resuscitation knowledge and skills. In contrast, healthcare professionals with low self-efficacy perceptions may experience uncertainties regarding their capabilities and demonstrate hesitancy regarding participating in critical and high-risk scenarios, such as cardiopulmonary resuscitation. Therefore, it is recommended to use educational methods that enhance self-efficacy perception during CPR training [15]. Scenario-based simulation training can be used to improve self-efficacy perceptions and reduce these difficulties.

In this study, a high-fidelity scenario-based simulation was conducted for intern students in their final year of medical education. The aim of this study was to investigate the effect of ALS simulation on students’ self- efficacy. The data revealed that the percentage of change in the CSES was significantly higher among students who participated in the ALS simulation than among those who did not (*p* < 0.05). (Table 4.)

Additionally, there are studies that emphasize the gap between clinicians’ self-efficacy perceptions and their actual level of competence [14]. A systematic review conducted by Davis et al. [27] emphasized the lack of consistent correlation between observed self-efficacy measurements of physicians and their actual self-efficacy, suggesting a limited ability for accurate self-assessment. Interestingly, they found that physicians with lower skills, but higher self-efficacy tended to have poorer self-evaluation abilities.

Gonzi et al. investigated the relationship between self-efficacy perception and performance by examining the correlation between hospital staff’s self-efficacy perceptions in basic life support and their actual performance during the application. The findings indicated that there was no significant correlation between the self-efficacy perception measured before the simulation and the performance measured during the application [28]. In a pediatric resuscitation study by Turner et al., participants’ performance in high- fidelity simulation training was evaluated as an indicator of the probability of transferring self-efficacy to clinical learning practice. However, no correlation was found between self-efficacy and resuscitation skills performance [29].

The relationship between performance and self-efficacy perception is a critical aspect to consider. Healthcare professionals may demonstrate suboptimal CPR performance [30]. It is hypothesized that physicians with high self-efficacy perceptions, but low performance may lack awareness of their deficiencies in meeting specific skill requirements, resulting in overconfidence. This may pose significant risks, especially in critical cases that require resuscitation [14]. The perception of healthcare professionals in evaluating the accuracy of their CPR skills may not be as accurate as the objective feedback methods used during CPR simulations. Additionally, this perception may lead to an overestimation of compression quality, which can result in low- quality CPR. The self-efficacy perceptions of healthcare professionals exhibited a stronger correlation with their actual performance following CPR trainings involving simulation. In addition, it was observed that the self-evaluation processes were better maintained under these conditions [28].

In our study, we assessed students’ self-efficacy perceptions using the ALS Technical Skills Assessment Tool, and their skills were also assessed by an observer during the simulation training. However, the compatibility analyses conducted using the obtained data revealed a discrepancy between the student’s self-efficacy perceptions and the observer’s performance scores. The correlation between the students’ ALS performances and their self-efficacy perceptions was low. Furthermore, we investigated the correlation between students’ self-efficacy perceptions regarding cardiac compression skills and their actual performance by using CPR feedback bracelets. The results demonstrated that the success rates of students in both components of this fundamental skill were remarkably low (Table 6). Despite the low success rates, students generally perceived themselves as self-sufficient. The CPR device measurements revealed low success rates, indicating an inconsistent relationship between self-efficacy perception and demonstrated performance.

The theme of “Self-efficacy Perception” during the focus group interviews revealed that the code of “recognition of individual insufficiency and inexperience” was expressed at a ratio of 28.57%. Participants made statements such as “I faced my insufficiency during the training and " For the first time, ALS management was entirely left to us, and we saw the benefits of this. There was an opportunity to see our mistakes.” It is great, of course, to be more competent. “I saw my mistakes and believe I won’t repeat them. I gained more confidence in myself regarding actually doing it.” “In this simulation, we not only witness our own development but also feel more self-assured”.

These statements emphasize the effectiveness of using simulation in training procedures to establish a connection between self-efficacy perception and performance. Students show higher scores and increased self-efficacy perceptions in the ALS training conducted with high-fidelity simulations. The use of simulation in ALS training enhances knowledge and self-efficacy. The debriefing step, which involves reflection and evaluation of performance during simulation scenarios, can reduce potential risks for patients. This step enhances self-efficacy perception related to individual performances.

## Conclusion

In conclusion, our study showed that students who participated in simulation showed a higher percentage of changes in self-efficacy scores than those who did not participate. The findings indicated that the simulation experience contributed to the development of students’ confidence in their ability to manage ALS independently This was supported by their increased perception of adequacy in ALS, confidence in future interventions, and acknowledgment of personal inadequacy and lack of experience.

During medical education, students face various challenges in their CPR training. Real- life ALS situations often involve a chaotic environment where students may not have the opportunity to fully practice each step of ALS. Moreover, mastering ALS requires the acquisition of numerous psychomotor skills. In addition, the ALS process includes non-technical skills such as self-efficacy belief, effective teamwork, leadership, crisis management, clinical decision-making, delivering bad news, and stress management, all of which are essential for successful resuscitation.

Developing mechanisms that effectively transfer these skills to real-life situations and provide frequent repetition using realistic scenarios can be challenging throughout the duration of medical education. In cases where there is a high perception of ALS self-efficacy but inadequate performance, considering the potential risks to the patient, it is crucial to design ALS training programs throughout medical education that promote high levels of both self-efficacy perception and performance, ensuring they are correlated. These training programs should be carefully designed and integrated into educational curricula with a learner- centered approach, focusing on the holistic development and acquisition of complex skills essential for patient safety. It is also important to regularly provide students with opportunities for self-assessment and evaluation of their performance and skill development in ALS.

It is evident that advanced life support simulations are more commonly investigated in studies involving healthcare professionals who are experts or experienced in this field. Unlike simulation research conducted with healthcare professionals competent in ALS, this study comprehensively examines the impact of high-fidelity scenario-based ALS simulations on medical students. It showcases a broader potential role of these simulations in under-graduate medical education and offers a perspective on health education.

## Data Availability

The data that support the findings of this study are not openly available due to reasons of sensitivity and are available from the corresponding author upon reasonable request. Data are located in controlled access data storage at Kocaeli University Faculty of Medicine.
